# Effect of national culture on BMI: a multilevel analysis of 53 countries

**DOI:** 10.1186/s12889-019-7536-0

**Published:** 2019-09-03

**Authors:** Mohd Masood, Akash Aggarwal, Daniel D. Reidpath

**Affiliations:** 1 Department of Dentistry and Oral Health, La Trobe Rural Health School, La Trobe University, Bendigo, Australia; 2 Division of Population and Patient Health, Dental Institute, King’s College London, London, UK; 3 Department of Global Public Health, Jeffery Cheah School of Medicine and Health Science, Monash University, Bandar Sunway, Malaysia

**Keywords:** Obesity, BMI, Culture

## Abstract

**Background:**

To investigate the association between national culture and national BMI in 53 low-middle- and high-income countries.

**Methods:**

Data from World Health Survey conducted in 2002–2004 in low-middle- and high-income countries were used. Participants aged 18 years and over were selected using multistage, stratified cluster sampling. BMI was used as an outcome variable. Culture of the countries was measured using Hofstede’s cultural dimensions: Uncertainty avoidance, individualism, Power Distance and masculinity. The potential determinants of individual-level BMI were participants’ sex, age, marital status, education, occupation as well as household-wealth and location (rural/urban) at the individual-level. The country-level factors used were average national income (GNI-PPP), income inequality (Gini-index) and Hofstede’s cultural dimensions. A two-level random-intercepts and fixed-slopes model structure with individuals nested within countries were fitted, treating BMI as a continuous outcome variable.

**Results:**

A sample of 156,192 people from 53 countries was included in this analysis. The design-based (weighted) mean BMI (SE) in these 53 countries was 23.95(0.08). Uncertainty avoidance (UAI) and individualism (IDV) were significantly associated with BMI, showing that people in more individualistic or high uncertainty avoidance countries had higher BMI than collectivist or low uncertainty avoidance ones. This model explained that one unit increase in UAI or IDV was associated with 0.03 unit increase in BMI. Power distance and masculinity were not associated with BMI of the people. National level Income was also significantly associated with individual-level BMI.

**Conclusion:**

National culture has a substantial association with BMI of the individuals in the country. This association is important for understanding the pattern of obesity or overweight across different cultures and countries. It is also important to recognise the importance of the association of culture and BMI in developing public health interventions to reduce obesity or overweight.

## Background

Globally in 2010, obesity accounted for approximately 3.4 million deaths, 3.9% of years of life lost, and 3·8% of disability-adjusted life-years [1, 2]. Outside the clinical and bench sciences, obesity-related research has mostly focussed on identifying individual-level and neighbourhood level factors that could explain the trends in increasing BMI observable around the world [3]. Little research has been conducted to identify country-level factors for the variation in BMI and that too mainly focussed on national income [4]. If variation in BMI levels across the countries can be associated with national income, it may also be associated with national cultural factors.

“Culture” has a myriad definition which is hotly contested within anthropology, and between anthropology and other disciplines [5]. One common set of definitions relate to shared beliefs, norms, and values transmitted across generations [6]. In *Social Causes of Health and Disease*, William Cockerham defined culture as: ways of living that have been passed on from one generation to the next in the form of abstract ideas, norms, habits, customs, and in the creation of material objects such as food, dress, housing etc. Culture thus refers to a body of common understandings that represent what groups of people and societies think, feel, and act upon. The knowledge, beliefs, values, customs, and behaviours shared by people in a particular society reflect the culture of that society [7].

Culture affects the circumstances in which we eat, the type of food we eat, with whom we eat it, the times of day we eat it, and the quantities we eat. Our dietary choices are patterned by biology, psychology, and economics. These choices reflect our cultures and our cultural identities [8]. Sociological and marketing studies underline how food represents an everyday materialization of ethnic identity, and resistance to make a change in food choices [9]. We would suggest that eating is culturally patterned, and by extension secular changes in population, adiposity will be influenced inter alia by the shared national culture of a population.

One challenge in attempting to explore culturally bound influences and their effect on obesity risk is the complexity inherent in measuring factors such as cultural values and beliefs [10]. For this reason, it is required to have quantifiable metrics for the culture that can provide comparable values for different cultures or societies or countries. Hofstede empirically developed a metric to measure national culture using four dimensions of countries’ culture. These dimensions are extensively validated against other aspects of national societies and for their cross-time stability [11]. It is the most comprehensive and robust framework in terms of the number of national cultures samples [12, 13]. Consequently, Hofstede’s operationalization of cultures (1984) is frequently used in research studies [14–17]. The aim of this study is to investigate the relationship between national culture using a valid national culture metric and body mass index.

## Methods

Data from the World Health Survey (WHS) conducted in 2002–2004 by the World Health Organization (WHO) in 70 countries was used in this study. The WHS was conducted for gathering valid, reliable and comparable information on health status and health system from low, middle and high-income countries. Adults aged ≥18 years living in private households in each nation were the target population for each nation. The target population, in each country, was adults aged ≥18 years living in private households. With the intentions of collecting nationally representative samples, multistage stratified cluster sampling was used to select participants. This project was approved by the Monash University Human Research Ethics Committee (MUHREC), Project Number: CF14/3907–2,014,002,034.

Individual-level BMI was estimated by using height and weight reported by the participant. Potential determinants of individual-level BMI considered in the analysis comprised various individual and country-level factors. Individual-level factors included: sex, age, marital status, education, occupation, economic status and location of household. These individual-level factors were selected based on the previous well established evidence for association of demographic factors like sex, age and marital status with BMI [2, 18–20]. Similarly, socioeconomic factors including education, occupation and economic status have been reported to influence both intake and expenditure of energy [19, 21]. Evidence for the location of household, rural or urban, and obesity is quite consistent as the prevalence of obesity in urban and rural areas has been reported to be higher in low and middle-income countries, and high-income countries, respectively [22–24]. Age was measured in years. Marital status could be married (including those living together), never married or formerly married (split, divorced or widowed). Educational status was classified into three levels: ≤ primary school, secondary school/college, or higher. A wealth index classifying households based on their occupancy of a range of household assets was used to determine the economic status of a household [25]. The household items included in the index were: the number of rooms, cars, chairs and tables in the house; the presence of electricity, bicycle, bucket, washing machine, dishwasher, refrigerator, fixed line telephone, mobile/cellular telephone, television, computer and clock [25]. Country-specific items according to living standards of the country were also included, and the final list comprised 11–20 items. Index of the asset variables for each country was then created based on the weights determined by principal component analysis (PCA). A continuous index measure was obtained by applying the weights of the first component to each individual’s data [25]. PCA score was then divided into five parts to define wealth quintiles as Quintile 1(poorest), Quintile 2 (lower-middle), Quintile 3 (middle), Quintile 4 (higher-middle), and Quintile 5 (wealthiest). Occupation was categorized following the Goldthorpe schema [26]: High (Legislator, Manager, Senior official, Professional and Armed Forces), medium (Technician, Associate Professional, Clerk, Service or sales worker), low (Agricultural, fishery worker, Craft, trades worker, Plant/machine operator or assembler) and elementary (elementary workers).

Country-level factors were average national income and income inequality as they have been most commonly used country-level economic factors in relation to health and obesity. National income was measured as GNI-PPP (centred at the mean of USD 8840) for the year 2003 [27]. Income inequality was determined using the Gini index, which varies from 0 (perfect equality) to 100 (perfect inequality) [28, 29]. Data on GNI-PPP and Gini index were obtained from the World Bank [27, 30].

National culture was measured using Hofstede’s cultural dimensions: Uncertainty avoidance (UAI), individualism (IDV), Power Distance (PDI) and masculinity (MAS). Data on these cultural dimensions was obtained from Hofstede’s book “Cultural Consequences”, 2nd edition [31]. Uncertainty Avoidance Index, Individualism index, Power distance index, and Masculinity versus Femininity Index from Hofstede et al. (2010) were referred for UAI, IDV, PDI and MAS scores, respectively [31]. These indices refer to relative differences between countries and scores varied between 0 and 100. Data on IDV, PDI, MAS and UAI was available only for 53 WHS countries. This paper analysed the relationship between national culture and BMI independent of physical activity.

Population estimates and standard errors for each country were generated using sampling weights to account for the stratification and clustering in the survey design. R-3.1.0 with the “survey package” was used for all design-based analyses and the lme4 package was used for multilevel linear regression analysis. A paper fitting the interaction term between individual level and country-level variables has been published previously [32].

In this study, we treated BMI as a continuous variable, and fitted a two-level random intercept and fixed-slopes model with individuals listed within countries. The full maximum likelihood method in R was applied to determine the fixed- and random-parameter estimates for the two-level regression model. Multilevel modelling incorporating survey design features are under continuous debate and are not currently available in R, therefore results from multilevel modelling were not weighted [33]. We first estimated the null model (model 0) and then gradually added explanatory variables into the model. All individual-level factors and GNI-PPP and Gini index were included as explanatory variables in Model 1. Cultural dimensions were subsequently added in the following models (model 2-model 5). In model 6, all the 3 significant dimensions were added together in the multivariate analysis to see the effect of all the cultural dimensions together.

## Results

**Fig. 1:**
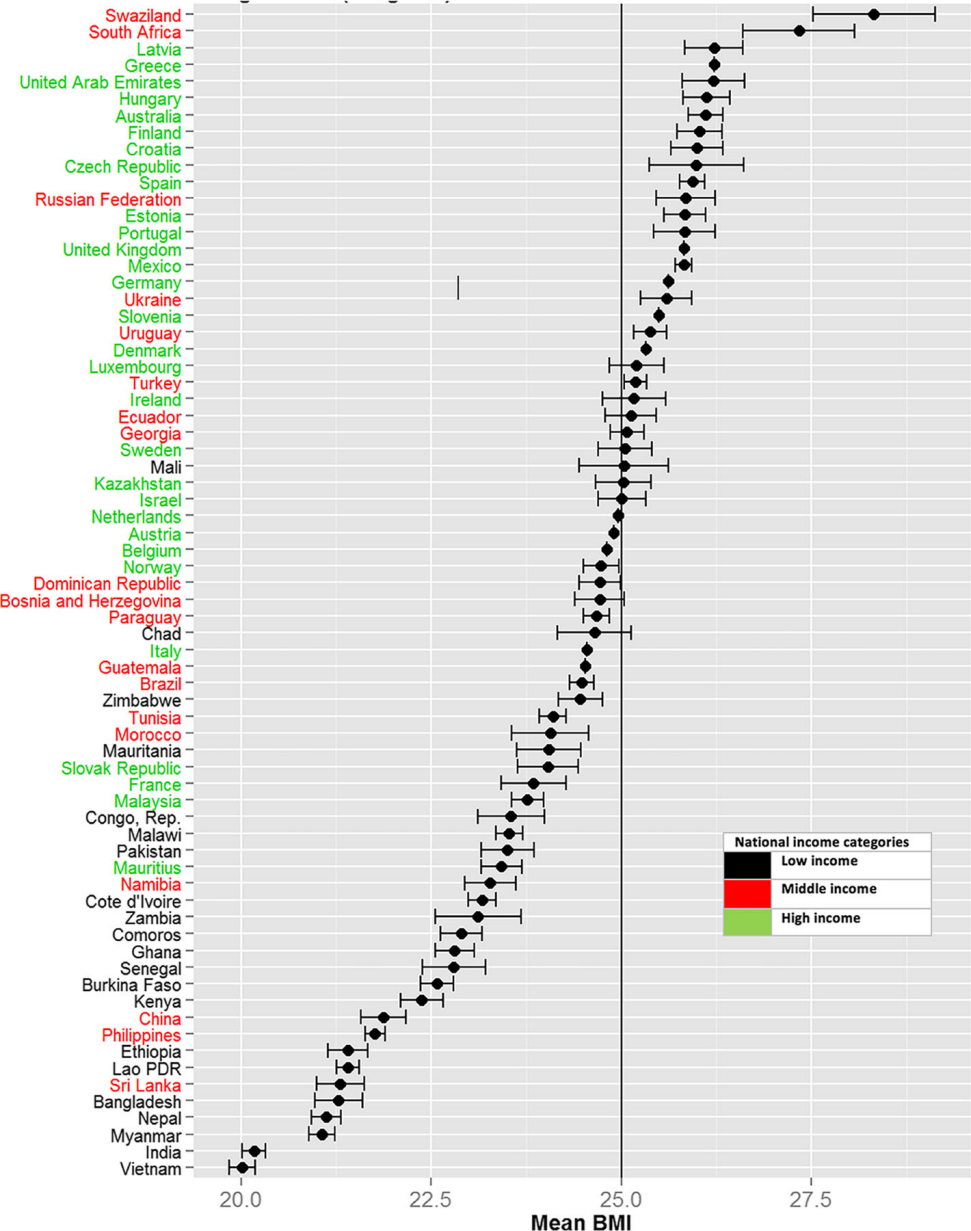
Design-based mean BMI (weighted) and confidence interval for each country

A sample of 156,192 people from 53 countries was included in this analysis (Table 1). The design-based (weighted) mean BMI (SE) in these 53 countries was 23.95(0.08) and the design-based (weighted) mean age (SE) of the sample from these 53 countries was 41.27(0.19) (Table 2). The pattern of mean BMI in 53 countries is presented in Fig. 1. The lower and higher end of the BMI were predominated by low-income countries and high or middle-income countries, respectively. Swaziland was an exception as a low-income country with a high BMI.

**Table 1:** Initial and final sample size after excluding values on height, weight and BMI variables

	Participants surveyed	Participants included in analysis	Response rate^a^
Australia	3600	2915	81.0
Austria	1055	948	89.9
Bangladesh	5552	856	15.4
Belgium	1012	956	94.5
Brazil	5000	4443	88.9
Burkina Faso	4825	1725	35.8
China	3993	3983	99.7
Croatia	990	980	99.0
Czech Republic	935	913	97.6
Denmark	1003	974	97.1
Dominican Republic	4534	3111	68.6
Ecuador	4660	4060	87.1
Estonia	1012	998	98.6
Ethiopia	4938	971	19.7
Finland	1013	1004	99.1
France	1008	951	94.3
Germany	1259	1180	93.7
Ghana	3938	3674	93.3
Greece	1000	961	96.1
Guatemala	4770	3193	66.9
Hungary	1419	1399	98.6
India	9994	9268	92.7
Ireland	1014	910	89.7
Israel	1236	1185	95.9
Italy	1000	958	95.8
Kenya	4417	4288	97.1
Latvia	856	735	85.9
Luxembourg	700	692	98.9
Malawi	5306	5185	97.7
Malaysia	6040	4989	82.6
Mexico	38,746	23,480	60.6
Morocco	5000	2041	40.8
Myanmar	5886	5881	99.9
Namibia	4250	3766	88.6
Nepal	8688	3166	36.4
Netherlands	1091	1085	99.5
Norway	984	958	97.4
Pakistan	6379	3449	54.1
Philippines	10,078	8149	80.9
Portugal	1030	896	87.0
Russian Federation	4422	3501	79.2
Senegal	3226	1681	52.1
Slovak Republic	2519	1793	71.2
Slovenia	585	571	97.6
South Africa	2352	1460	62.1
Spain	6364	6161	96.8
Sri Lanka	6732	5663	84.1
Sweden	1000	975	97.5
Turkey	11,220	8149	72.6
United Arab Emirates	1180	1132	95.9
United Kingdom	1200	1059	88.3
Uruguay	2991	2965	99.1
Vietnam	3492	3475	99.5
Zambia	3812	2212	58.0

^a^Response rate after excluding missing and invalid values for height, weight and BMI

**Table 2:** Model based and design-based descriptive analysis of outcome variable (BMI) and individual-level explanatory variables in 53 countries

	Model Based	Design-based
	*n* = 156,192	*N* = 770,151,380
	Mean ± SD	Mean ± SE
Outcome variable		
BMI	24.05 (4.92)	23.95 (0.08)
Explanatory Variables		
Age	42.33 (16.71)	41.27 (0.19)
	n (%)	N (%)
Gender		
Female	71,876 (53.9)	3,861,707 (50.2)
Male	61,389 (46.06)	3,839,769 (49.8)
Missing values	5 (0.003)	3802 (0.0)
Education		
Primary school	53,122 (39.86)	351,559,014 (45.6)
Secondary school	64,018 (48.08)	304,854,666 (39.6)
College and above	15,041 (11.28)	109,509,803 (14.2)
Missing values	1026 (0.76)	4,227,898 (0.5)
Marital Status†		
Never Married	24,270 (18.21)	156,329,916 (20.3)
Married	74,971 (56.25)	459,772,891 (59.7)
Widowed/Divorced	25,499 (19.13)	122,482,578 (15.9)
Missing values	8530 (6.4)	31,565,995 (4.1)
Household Income		
1st Quintile (Poorest)	26,030 (19.53)	155,540,304 (20.2)
2nd Quintile	26,196 (19.65)	151,537,449 (19.7)
3rd Quintile	24,542 (18.41)	137,002,987 (17.8)
4th Quintile	24,592 (18.45)	140,199,329 (18.2)
5th Quintile (Wealthiest)	24,267 (18.20)	12,525,755,316.3)
Missing values	7643(5.73)	60,613,759(7.9)
Occupation‡		
High	10,090(7.57)	56,431,105(7.3)
Medium	18,797(14.10)	106,090,097(13.8)
Low	31,012(23.27)	212,328,723(27.6)
Elementary	6658(4.99)	39,368,661(5.1)
Missing values	66,713(50.05)	355,932,795(46.2)
Setting¥		
Urban	75,102(56.35)	355,475,737(46.2)
Rural	52,265(39.21)	386,726,171(50.2)
Missing values	5903(4.42)	27,949,472(3.6)

†All data in this variable was missing for Turkey; ‡All data in this variable was missing for Turkey and Norway; ¥ All data in this variable was missing for Australia, Netherlands, Norway and Slovenia; Design-based- probability of selection design weights; N-target population; SD- Standard Deviation; SE- Standard Error; BMI- Body Mass Index. ^Ψ^Occupation categories: High (Legislator, Senior Official, or Manager, Professional and Armed Forces), Middle (Technician or Associate Professional, Clerk, Service or Sales Worker), Low (Agricultural or fishery worker, Craft or Trades Worker, Plant/machine Operator or Assembler), and Elementary (Elementary Workers)

Results of multilevel models for BMI and country-level and individual-level variables are presented in Table 3. First, we ran the null model or the variance component model for 53 countries. The fixed part is represented by the coefficient for the constant, which is 24.60 with a standard error of 0.25. That is to say, the estimated overall population mean for BMI is 24.60 for 53 countries. The random part is given under the heading “Random effect” for the variance of level 1 residuals and “variance and covariance of random effects” for the variance of the random intercept. Accordingly, the estimate of the between-countries variance is 2.82 (SD = 1.68) and the estimate of within country variance is 20.41 (SD = 4.52). A total of 12.0% of variance of BMI can be explained by the variations in the characteristics of countries as suggested by the intra-class correlation (proportion of total variance occurring between countries) of 0.12 for BMI in these countries.

**Table 3:** Multilevel multivariate linear regression analysis with individual and country level predictors in 53 countries

	Model 1		Model 2		Model 3		Model 4		Model 5	
	β	SE	β	SE	β	SE	β	SE	β	SE
Fixed Effect										
Intercept	23.3	0.26***	23.3	0.24***	23.5	0.26***	23.5	0.26***	23.2	0.26***
Country Level										
GNI-PPP/10000	0.51	0.14***	0.44	0.13**	0.41	0.13**	0.45	0.13**	0.51	0.13**
Gini	0.05	0.03	0.03	0.02	0.07	0.02**	0.06	0.02*	0.05	0.02
UAI			0.03	0.009**						
IDV					0.03	0.009*				
PDI							- 0.02	0.009*		
MAS									0.005	0.009
Individual-level										
Age	0.04	0.001***	0.04	0.001***	0.04	0.001***	0.04	0.001***	0.04	0.001***
Gender										
Female	Reference category									
Male	0.012	0.03	0.012	0.03	0.012	0.03	0.012	0.03	0.012	0.03
Education										
Primary school	Reference category									
Secondary school	0.19	0.03***	0.19	0.03***	0.19	0.03***	0.19	0.03***	0.19	0.03***
College and above	−0.11	0.05*	−0.11	0.05*	−0.11	0.05*	−0.11	0.05*	−0.11	0.05*
Marital Status										
Never Married	Reference category									
Married	1.16	0.04***	1.16	0.04***	1.16	0.04***	1.16	0.04***	1.16	0.04***
Single	0.74	0.04***	0.74	0.05***	0.74	0.05***	0.74	0.05***	0.74	0.05***
Household Income										
1st Quintile (Poorest)	Reference category									
2nd Quintile	0.18	0.039***	0.18	0.039***	0.18	0.039***	0.18	0.039***	0.18	0.039***
3rd Quintile	0.30	0.039***	0.30	0.039***	0.30	0.039***	0.30	0.039***	0.30	0.039***
4th Quintile	0.38	0.039***	0.38	0.039***	0.38	0.039***	0.38	0.039***	0.38	0.039***
5th Quintile (Wealthiest)	0.50	0.039***	0.50	0.039***	0.50	0.039***	0.50	0.039***	0.50	0.039***
Occupation^Ψ^										
High	Reference category									
Middle	−0.043	0.057	−0.043	0.057	−0.043	0.057	−0.04	0.057	−0.043	0.057
Low	− 0.25	0.057***	− 0.25	0.057***	− 0.25	0.057***	− 0.25	0.057***	− 0.25	0.057***
Elementary	0.16	0.074*	0.16	0.074*	0.16	0.074*	0.16	0.074*	0.16	0.074*
Setting										
Urban	Reference category									
Rural	−0.36	0.03***	−0.36	0.03***	− 0.36	0.03***	−0.36	0.03***	−0.36	0.03***
Random effect										
Country	1.8	1.34	1.5	1.23	1.54	1.23	1.62	1.27	1.77	1.33
Residual	19.60	4.41	19.60	4.43	19.60	4.43	19.60	4.43	19.60	4.43
Fit Indices										
AIC	775,010.0		775,004.2		775,005.4		775,007.5		775,011.7	
BIC	775,225.6		775,229.6		775,230.8		775,232.9		775,237.1	
Log Likelihood	− 387,483.0		−387,479.1		−387,479.7		−387,480.8		−387,482.9	
Deviance	774,966.0		774,958.2		774,959.4		774,961.5		774,965.7	
Model Comparison	With model 0		With model 10		With model 12					
Chi-sq (df)	5465.3(19)***		10.28(1)**		6.6(1)*		4.5(1)*		0.30(1)	
R^2^										
Country Level R^2^	0.362		0.468		0.454		0.426		0.372	
Individual-level R^2^	0.040		0.040		0.040		0.040		0.040	
Total R	0.079		0.092		0.090		0.087		0.080	

*p value≤0.05; **p value≤0.01; ***p value≤0.001; *β* regression coefficient, *SE* Standard Error, *AIC* Akaike information criterion, *BIC* Bayesian information criterion, *Chisq* Chi Square test, *df* Degree of freedom; GNI-PPP/10000- National income; Gini- income inequality; UAI-Uncertainty Avoidance; IDV- Individualism, PDI- Power distance, MAS- Masculinity; ^Ψ^Occupation categories: High(Legislator, Senior Official, or Manager, Professional, and Armed Forces), Middle(Technician or Associate Professional, Clerk, Service or Sales Worker), Low (Agricultural or Fishery Worker, Craft or Trades Worker, Plant/machine Operator or Assembler), and Elementary (Elementary Workers)

In Model 1 the combined effect of all individual-level variables, GNI-PPP and Gini were tested on BMI (Table 3). There was a positive association between age and BMI, 0.34 units increase in BMI for every 10 years increase in age. However, there was no significant association between gender and BMI. On average people with primary education had lower BMI than those with secondary education. BMI for the married group was significantly higher as compared to never married and previously married groups. A significant association between household wealth and BMI was also found. All wealthier quintiles had higher BMI than the lowest quintile when the rest of the variables are kept constant. Occupation variable showed that BMIs for professionals and elementary workers were not significantly different. However, the mean BMI for people with low occupation was significantly lower than that for professionals. Similarly, mean BMI for people living in rural areas was significantly lower than people living in urban areas. GNI-PPP (β = 0.51, *p* < 0.001) was positively related but the Gini index did not have any relationship with BMI.

In following models, four cultural dimensions were added one by one to see independent effect of each dimension. UAI (β = 0.03, p < 0.001) was significantly associated with BMI (model 3). IDV (β = 0.03, *p* < 0.001) was also significantly associated, showing that people are heavier in more individualistic countries than less individualistic (collectivist) ones. In this model, regression coefficient for Gini index becomes significant (β = 0.07, *p* < 0.05). PDI was also significantly associated with BMI, each unit increase in PDI was associated with 0.02 unit decrease in BMI. MAS was not significantly related to BMI.

In final model Table 4, all the 3 significant dimensions were added together in the multivariate analysis to see the effect of all the cultural dimensions together. This model explained that one unit increase in UAI or IDV was associated with 0.03 unit increase in BMI. However, the effect of PDI on BMI disappeared in this model. It means that the PDI in a country does not have any effect on an individual’s BMI after considering the country’s UAI and PDI. Relationship of Gini index (β = 0.06, *p* < 0.01) with BMI got stronger in this model after considering cultural dimensions. This final model explained 61.7% of country-level and 11.0% total variance in BMI.

**Table 4:** Multilevel multivariate linear regression analysis with individual and country level predictors in 53 countries

	Model 6	
	β	SE
Fixed Effect		
Intercept	23.6	0.23***
Country Level		
Log GNI-PPP/10000	0.30	0.14*
Gini	0.06	0.02**
Uncertainty avoidance	0.03	0.008***
Individualism	0.03	0.01*
Power Distance	−0.01	0.009
Individual-level		
Age	0.04	0.001***
Gender		
Female	Reference category	
Male	0.012	0.03
Education		
Primary school		
Secondary school	0.19	0.03***
College and above	−0.11	0.05*
Marital Status		
Never Married	Reference category	
Married	1.16	0.04***
Single	0.74	0.04***
Household Income		
1st Quintile (Poorest)	Reference category	
2nd Quintile	0.18	0.039***
3rd Quintile	0.30	0.039***
4th Quintile	0.38	0.039***
5th Quintile (Wealthiest)	0.50	0.039***
Occupation ^Ψ^		
High	Reference category	
Middle	−0.044	0.057
Low	−0.25	0.057***
Elementary	0.16	0.074*
Setting	Reference category	
Urban		
Rural	−0.36	0.03***
Random effect		
Country	1.08	1.04
Residual	19.60	4.41
Fit Indices		
AIC	774,995.3	
BIC	775,250.1	
Log Likelihood	−387,471.7	
Deviance	774,943.3	
Model Comparison	With model 0	
Chi-sq (df)	22.6(4)***	
R^2^	With model 0	
Country Level R^2^	0.617	
Individual-level R^2^	0.040	
Total R	0.11	

*p value≤0.05; **p value≤0.01; ***p value≤0.001; β- regression coefficient; SE- Standard Error; AIC- Akaike information criterion; BIC- Bayesian information criterion; Chisq- Chi Square test; df- Degree of freedom; ^Ψ^Occupation categories: High(Legislator, Senior Official, or Manager Professional, and Armed Forces), Middle(Technician or Associate Professional Clerk, Service or Sales Worker), Low (Agricultural or Fishery Worker, Craft or Trades Worker, Plant/Machine Operator or Assembler), and Elementary (Elementary Workers)

## Discussion

This study found that UAI and IDV had a significant positive association with BMI in 53 WHS countries after controlling for other cultural dimensions, national income, income inequality and individual-level factors. People from high individualistic or high uncertainty avoidance countries had higher BMI compared with people from low individualistic or low uncertainty avoidance countries. This observed association warrants the exploration of the differences in the characteristics of low and high individualistic or uncertainty avoidance countries. Following some possible characteristics of such countries are discussed to explore this observed association.

In high uncertainty avoidance cultures, it is expected that individuals engage in careful planning to reduce risks by attempting to control future events [34, 35]. In this scenario, it is expected that the people from high uncertainty avoidance countries should have planned for the uncertainty related to obesity and related health outcomes and should have more strict rules and regulations related to those issues to prevent or reduce it. This argument indicates a negative association between uncertainty avoidance and BMI but results in this study showed a reverse pattern. There are a few reasons for this reverse pattern of high BMI in high uncertainty avoidance, countries. Paradoxically, in countries with weak uncertainty avoidance where rules are less sacred, they are often better followed. However, in countries with strong uncertainty avoidance, laws can fulfil a need for security, even when they are not followed [28]. Additionally, these high uncertainty avoidance countries usually plan for future ambiguous situations related to obesity, mainly by planning for curative treatment with more specialists and utilization of more medicine [17, 36]. Tolerance to familiar risks has been reported to be very high in UAI countries [37, 38]. As obesity and overweight are encountered on a regular basis, tolerance to familiar risk activities which predispose to these problems is likely to be high. It is more challenging to instil ownership of obesity prevention when the problem is regarded as a countrywide issue, rather than a country in which relatively less percentage of the population has obesity. In such situations, there is a greater likelihood of non-compliance of key preventive strategies and interventions, such as physical activity, which require extra effort or time [39]. Moreover, once a behaviour, healthy or unhealthy, is adopted it is difficult to use new policies to change this behaviour due to instinctive resistance to change [40]. In cultures with high UAI, people expect health professionals or the government to provide solutions for the problems and expect that the experts always have a solution [41, 42]. This leads to a more curative rather preventive attitude in people towards obesity [43]. It is expected that people are likely to find difficulty in accepting a recommendation to prevent or manage obesity simply through healthy diet management and more physical activities. Public health approaches to prevent or control obesity in a country must consider its’ UAI dimension. The clarity in the message or in the content is strongly desired in high UAI countries [37]. High level verbal specificity is required to maintain the sense of security in one’s beliefs, and communication that includes free verbal play with its inevitable risks of misunderstanding should be avoided. To implement a public health policy or programme in a country with higher UAI scores, the proposals should be backed up with facts and statistics to negate uncertainty, it should not be expected that unfamiliar policies, ideas or methods will be readily embraced [44]. Enough time should be allowed to help people to develop an understanding of the initiative to help foster confidence in it; community involvement in projects is desired to develop a sense of understanding, and then decrease the element of the unknown [36, 45].

Low IDV (collectivist) societies believe that health is controlled by external sources beyond their control such as the family, society. Cheng et al. (2013) described in a meta-analysis that in collectivist societies decision making and group behaviour are largely determined by the contexts such as society or family [46]. Families tend to eat together, portion sizes are reduced, and snacking behaviour is less frequent [36, 47, 48]. In contrast, members of individualistic societies tend to consider the decision-making and individual behaviour to be contingent upon their own actions, under personal control, and relatively independent of the contexts such as society or family. This personal control on the food is associated with higher intake of food, larger portions of food, no one to share with, and more frequent snacking probably due to more snacking opportunities [49, 50]. For example, in most individualist countries such as the UK and the USA, the concept of ‘children’s food’ has developed. In most other cultures, young children gradually move from a diet of baby food to family food. But in these individualist countries, there are certain types of food (fish fingers, baked beans, chicken nuggets) which are specifically designed and marketed for children [51]. This availability of genre of children food has two consequences. At first, as this is children’s food, children have more control over the size of the serving. Secondly, children have become customers in their own right for manufactures selling easy to prepare foods of poor nutritional value which may be in part a contributing factor for increased BMI levels [52].

The motivations for eating and physical activity also vary among different cultures [37]. In a collectivist society, like Japan, physical and environmental motivations are usual triggers for eating. Physical eating is triggered by hunger cues, like growling stomach or the feeling of dizziness. Environmental eating occurs in response to something in the surroundings, such as hearing the lunch bell, smell of food, or check-out stands. People in an individualistic society are triggered to eat, without having specific feelings of hunger or nutritional needs, based on their emotional status and environmental cues such as out of boredom, watching TV or movies [53].

Members of a collectivist society spend more time in role or context dictated activities, which include tending animals, gardening, sleeping, cooking and eating. In contrast, people from individualist societies spend most of their time in idle leisure activities e.g. watching TV, internet and reading papers [36, 54]. For people in individualist societies, lack of motivation is the most commonly reported obstacle to healthy eating and physical activity. For example, residents of an individualistic society such as the USA tend to have high expenditures on amenities which make their lives easier and reduce exercise or effort [50].

Third, the response rate has varied considerably across the countries. However, most of the countries included in this study had good response rates of more than 60%, except Bangladesh and Ethiopia. Achieving high response rates in national surveys is always challenging, especially for low and middle-income countries. Nonetheless, the results of this study should be interpreted considering the inherent selection bias secondary to exclusion of probability of selection weights from random effects model. Fourth, there are different cut-off values of BMI for obesity and overweight has been suggested based on the different geographic region and ethnicity. These different cut-off points make a multi-country comparison of overweight and obesity more challenging as the country (or ethnic) specific cut-off points for all countries (or ethnic groups) are not available to get correct overweight and obesity prevalence for each country [55]. Therefore, the Body Mass Index (BMI) as a continuous outcome variable is used in this study. Fifth, The WHS data was collected in 2002–2004 and might put uncertainty about the validity of results after 15 years. The main reason for using WHS datasets for this study was that these are unique comparable datasets available for 70 countries representing the countries from a range of low, middle and high-income countries.

## Conclusion

Culture affects the circumstances in which we eat, the types of food we eat, with whom we eat it, the times of day we eat it, and the quantities we eat. It is important to understand the relationship between culture and BMI. This study showed national culture has a substantial association with BMI of the individuals in the country. Uncertainty-avoidance and individualism national cultural dimensions were significantly associated with BMI, showing that people are heavier in more individualistic or high uncertainty-avoidance countries. This association is important for understanding the pattern of obesity or overweight across different cultures and countries. It is also important to recognise the importance of the association of culture and BMI in developing public health interventions to reduce obesity or overweight.

## Data Availability

Data used in this is publicly available and can be accessed from World Health Survey section of the World Health Organization website. http://apps.who.int/healthinfo/systems/surveydata/index.php/catalog/whs
